# Recharging the Powerhouse: Mitochondrial Dysfunction and Therapy in Cardiorenal Syndrome Type 4

**DOI:** 10.1007/s11897-025-00713-0

**Published:** 2025-09-13

**Authors:** Edouard Long, Joshua M. Heihre

**Affiliations:** 1 Faculty of Life Sciences and Medicine, King’s College London, London, UK; 2 Institute of Cardiovascular Science, University College London, London, UK; 3 Department of Clinical Neurosciences, University of Cambridge, Cambridge, UK

**Keywords:** Cardiorenal syndrome, Mitochondria, Heart failure, Chronic kidney disease, Inflammation, Oxidative stress

## Abstract

**Purpose of Review:**

Cardiorenal syndrome type 4 (CRS-4) is characterised by the development of cardiac dysfunction secondary to chronic kidney disease (CKD). This review outlines the pathophysiology of CRS-4, with a focus on the emerging role of mitochondrial dysfunction, and evaluates novel mitochondria-targeting therapeutics for CRS-4.

**Recent Findings:**

Current research has positioned mitochondrial dysfunction in cardiomyocytes as a key driver of CRS-4 pathophysiology, characterised by impaired adenosine triphosphate production, increased reactive oxygen species (ROS) generation, dysregulated mitophagy, altered mitochondrial biogenesis and dynamics, and bioenergetic malfunction. Currently licensed drugs, such as dapagliflozin and sacubitril/valsartan, have demonstrated mitoprotective effects in CRS-4, and numerous other therapies targeting mitochondria have proven efficacious in preclinical studies. However, real-world clinical trials are required to determine whether mitochondria represent a viable therapeutic target that offers meaningful clinical benefits to patients with CKD.

**Summary:**

There is increasing evidence that mitochondrial dysfunction is a key pathomechanism in the development of CRS-4. Mitochondrial-targeting therapies offer a novel mechanism-driven approach, with numerous showing preclinical promise. However, real-world clinical trials are required to determine their therapeutic potential.

## Introduction

The link between renal and cardiovascular disease was first described by Robert Bright in the 1800s [1], since when a more nuanced understanding of the cardio-renal axis has led to the classification of five different types of ‘cardiorenal syndrome’ (Table 1) [2]. Type 4 cardiorenal syndrome (CRS-4), also known as chronic renocardiac syndrome, is characterised by the development of cardiovascular disease secondary to chronic kidney disease (CKD) [2]. CKD most commonly arises from diabetes mellitus (DM), followed by glomerulonephritis and hypertension [3], and can be classified into five stages based on glomerular filtration rate (GFR) and three levels according to albuminuria [4]. Patients with CKD have a 10- to 20-fold greater risk of cardiovascular death compared to age- and sex-matched cohorts without CKD [2] due to a number of factors, including structural heart disease [5, 6], arrhythmias [7, 8], and coronary heart disease [9]. There is emerging evidence that mitochondria play a key role in the pathogenesis of CRS through alterations of key mitochondrial processes, including biogenesis [10], mitophagy [11], and dynamics [12] alongside excessive production of reactive oxygen species (ROS) [13] and bioenergetic malfunction [14]. Increased understanding of these mechanisms has spurred the development of novel therapeutics that target these processes and may provide novel treatments for this group of patients for whom few efficacious therapies currently exist [15]. This review will outline the pathophysiology of CRS-4 and discuss the role of cardiac mitochondrial dysfunction, alongside novel therapies targeting these pathomechanisms.

**Table 1:** Classification of cardiorenal syndrome

CRS Type	Definition	Possible causes
CRS type 1 (acute CRS)	Development of an acute kidney injury due to a rapid decline in cardiac function.	- Cardiogenic shock - Acute chronic heart failure exacerbation - Acute coronary syndrome
CRS type 2 (chronic CRS)	Development of chronic kidney disease secondary to a chronic decline in cardiac function.	- Chronic heart failure with preserved/reduced ejection fraction
CRS type 3 (acute renocardiac syndrome)	Acute cardiac dysfunction secondary to an acute and primary decline in renal function.	- Renal ischaemia - Acute kidney injury - Glomerulonephritis
CRS type 4 (chronic renocardiac syndrome)	Development of decreased cardiac function due to primary chronic kidney disease.	- Chronic kidney disease e.g., chronic glomerular disease
CRS type 5 (secondary CRS)	Combined renal and cardiac dysfunction secondary to systemic disorders.	- Sepsis - Diabetes - Amyloidosis - Cirrhosis

As defined by Ronco et al. [2]

Abbreviations: CRS – cardiorenal syndrome

## Pathophysiology of CRS Type 4

The pathophysiology of CRS-4 is complex and multifactorial in nature. CKD-induced mechanisms contributing to the development of cardiovascular disease include CKD mineral and bone disorder (CKD-MBD), cardiorenal anaemia syndrome (CRAS), dyslipidaemia, cardiac arrhythmias, neurohormonal activation, pressure and volume overload, uraemic toxins, and oxidative stress [13, 16–22].

It should be noted that although the CKD population has a greater than average burden of baseline cardiovascular risk, these risk factors alone are not able to account for the greater incidence of cardiovascular disease [23–25]. This was illustrated by Matsushita et al. who meta-analysed over 600,000 individuals and found that eGFR and albuminuria were associated with cardiovascular outcomes independently of risk factors such as diabetes, blood pressure, and age [26]. This demonstrates the existence of other mechanisms, specific to CRS, which account for this increased cardiovascular risk.

CKD-MBD is characterised by the disruption of skeletal homeostasis due to disordered calcium-phosphate metabolism. In healthy individuals, fibroblast growth factor-23 (FGF23) is produced by osteocytes secondary to phosphate and/or vitamin D loading, and promotes urinary phosphate excretion alongside decreased circulating vitamin D concentrations [27, 28]. In CKD, as renal phosphate excretion worsens, a compensatory response can cause FGF23 levels to increase [28]. Increased FGF23 levels can then cause left ventricular hypertrophy (LVH), through activation of calcineurin–nuclear factor of activated T-cells (NFAT) signalling [27]. Raised FGF23 is independently associated with increased cardiovascular mortality [29]. The adverse cardiac implications of elevated FGF23 levels were demonstrated by Patel et al. in a cohort of over 2,000 patients over 10 years of follow-up [30]. In their study, elevated FGF23 at baseline was independently associated with greater LV mass and impaired LV strain. Interestingly, FGF23 was also associated with lower left atrial (LA) total emptying fraction which suggests that FGF23 may cause LA-specific cardiac damage and therefore increase susceptibility to atrial fibrillation (AF).

In CRS-4, anaemia is predominantly due to depressed erythropoietin (EPO) production secondary to CKD and damage to peritubular fibroblasts [31]. However, there are other factors which have been implicated including iron deficiency, disruption of erythropoiesis by uraemic toxins, CKD-MBD, reduced red blood cell lifespan, and treatment with renin-angiotensin-aldosterone inhibitors [32]. Anaemia can cause chronic hypoxia leading to reduced oxygen delivery to the myocardium, ventricular hypertrophy, and eventually impaired systolic function [17].

The development of cardiac arrhythmias in CRS-4 is multifactorial and can be a result of imbalances in electrolyte homeostasis, dialysis-induced arrhythmias, and CRS-induced LVH [20, 33, 34]. Patients with severe CKD may require dialysis, which in itself is a highly arrhythmogenic procedure due to the rapid changes in electrolytes, particularly potassium, that it induces [33]. This is demonstrated by the fact that sudden cardiac death is the most common cause of death among dialysis patients [34]. CKD-induced myocardial fibrosis can also promote anatomical re-entry pathways, which subsequently trigger ventricular arrhythmias [20]. There is also evidence that AF is significantly increased in CKD patients. Kim et al. Analysed over 4,500,000 patients over a median follow-up of 8.1 years and found that CKD was a significant, independent predictor of AF [35]. Compared to patients without CKD, adjusted hazard ratios for AF occurrence were 1.77 (95% confidence interval [CI]): 1.69–1.85), 1.85 (95% CI: 1.80–1.91), 1.99 (95% CI: 1.95–2.04), And 4.04 (95% CI: 3.07–5.33), for individuals with CKD stages 1–4, respectively. Epidemiological studies have estimated that as many as 30% of deaths in end-stage renal disease are secondary to arrhythmias or sudden cardiac death, which stresses the burden of arrhythmias in this population of patients [36].

The neurohormonal axis in renal homeostasis is predominantly controlled by the renin-angiotensin-aldosterone system (RAAS) alongside the sympathetic nervous system (SNS). In CKD, sodium and water retention cause increases in plasma volume, causing increased activity of RAAS and SNS. The resultant increase in angiotensin II (AngII) production can further promote fluid retention via aldosterone, initiating a vicious cycle [37]. RAAS-induced hypertension, alongside increases in arterial tone from sustained sympathetic activity and AngII production, increases cardiac afterload, augmenting cardiac wall stress and myocardial oxygen demand, which can ultimately cause LVH and systolic dysfunction [38].

The accumulation of uraemic toxins in CKD leads to the development of a ‘uraemic milieu’ which can subsequently induce uraemic cardiomyopathy. Uraemic cardiomyopathy encompasses a state of pathological cardiac remodelling characterised by diffuse fibrosis and LVH [39]. The mechanism behind this is complex and depends on the specific toxin. A number of uraemic toxins have been identified, which can be broadly split into protein-bound solutes, middle molecules, and free-water low-molecular-weight solutes [40]. Uraemic toxins can also induce vascular endothelial dysfunction via increased oxidative stress, which can then promote the development of atherosclerosis [41]. Others have also demonstrated that the uraemic milieu can trigger activation of the mitogen-activated protein kinase (MAPK), nuclear factor (NF)-*κ*B, and cyclic GMP-AMP synthase (cGAS)-stimulator of interferon genes (STING) pathways which can promote cardiac hypertrophy [41, 42]. It has also been proposed that dysregulation of neuregulin-1β (NRG-1β) signalling, a paracrine transmembrane growth factor essential in cardiac embryonic development, may be induced by uraemic cardiomyopathy and cause LVH and fibrosis [43]. NRG-1β signalling occurs via the erythroblastic leukaemia viral oncogene homologue (ErbB) family of tyrosine kinase receptors, which bind many growth factors. Sárközy et al. demonstrated that NRG-1β protein levels were decreased in CKD hearts with uraemic cardiomyopathy compared to controls. Subsequently, they demonstrated that administration of recombinant human NRG-1β prevented progression to LVH, suggesting that NRG-1β downregulation is a causal factor in the development of CRS-4 secondary to the uraemic milieu [43].

Oxidative stress, characterised by an imbalance between ROS production and antioxidant defences, plays a key role in the pathophysiology of CRS-4. In parallel, decreased nitric oxide (NO) synthesis, associated with endothelial dysfunction, further contributes to disease progression. ROS production can be stimulated by a variety of factors, including uraemic toxins and AngII [44]. The cardiotoxic sequelae of oxidative stress include the development of atherosclerosis and LVH [44]. The exact mechanism by which oxidative stress can induce LVH in CRS-4 remains unclear but there is experimental evidence that nicotinamide adenine dinucleotide phosphate oxidase (NOX) activity is increased, which subsequently activates the extracellular signal-regulated kinase 1/2 (ERK1/2) pathway and upregulates fibroblast growth factor‐2 (FGF2), a known promoter of cardiac fibrosis. Liu et al. analysed cardiac tissue from CRS-4 patients and found increased levels of superoxide dismutase (SOD), an antioxidant enzyme upregulated as a compensatory response to oxidative stress [44]. This elevated SOD activity reflects an underlying redox imbalance in cardiomyocytes, suggesting increased oxidative stress compared to controls. They then demonstrated in a rat model of CRS-4 that administration of apocynin, an antioxidant, reduced cardiac fibrosis via inhibition of the NOX-dependent ERK1/2 pathway, suggesting a causal role for ROS in the development of CRS-induced cardiac failure.

### Mitochondrial Pathophysiology in CRS-4

**Fig. 1:**
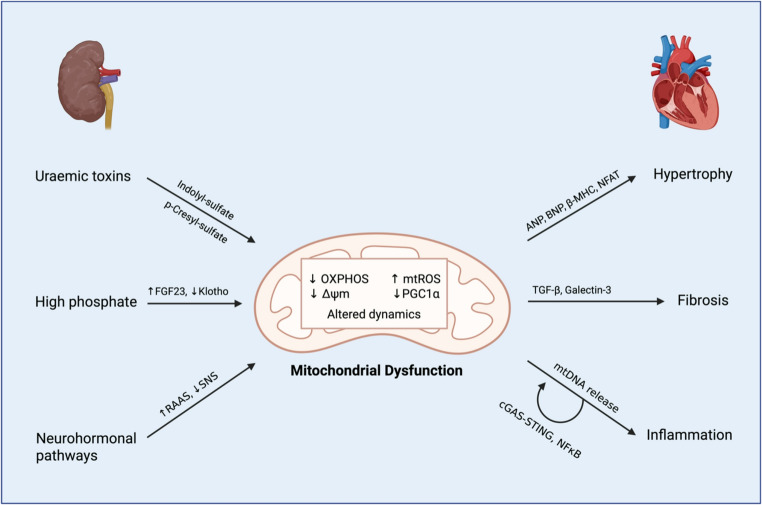
Summary of mitochondrial pathophysiology in CRS 4. CKD results in the release of uraemic toxins (such as indoxyl sulfate and p-cresyl-sulfate), raised phosphate levels (resulting in increased FGF23 and decreased Klotho), and neurohormonal (RAAS and sympathetic) activation. These cause mitochondrial dysfunction, which encompasses bioenergetic failure (↓ OXPHOS and ↓ ATP), excessive ROS production, loss of membrane potential (Δψm), and altered dynamics. This results in cardiomyocyte hypertrophy, manifesting at the organ level as concentric left ventricular hypertrophy and dysfunction. Abbreviations: CRS-4 – cardiorenal syndrome type 4, FGF23 – fibroblast growth factor-23, RAAS – renin-angiotensin-aldosterone system, SNS – sympathetic nervous system, OXPHOS – oxidative phosphorylation, mtROS – mitochondrial reactive oxygen species, PCG1a – proliferator-activated receptor gamma co-activator-1-alpha-1, ANP – atrial natuiretic peptide, BNP – brain naturetic peptide, b-MHC– beta-myosin heavy chain, NFAT – nuclear factor of activated T-cells, TGF-b – transforming growth factor beta, mtDNA – mitochondrial DNA, cGAS – cyclic GMP-AMP synthase, STING – stimulator of interferon genes, NF – nuclear factor

As the understanding of CRS-4 has progressed, mitochondria have emerged as potential mediators of the link between CKD and cardiovascular disease. Central mitochondrial processes dysregulated in cardiomyocytes during CRS-4 include impaired biogenesis, altered dynamics, defective mitophagy, increased generation of mitochondrial ROS (mtROS), and bioenergetic failure in the form of dysfunctional oxidative phosphorylation (OXPHOS) and decreased adenosine triphosphate (ATP) production [10–14]. These are summarised in Fig. 1.

The human mitochondrial genome consists of 16,569 base pairs, and mitochondrial DNA (mtDNA) lies within the mitochondria as a closed circular double-stranded molecule [45]. Notably, the heart possesses the greatest abundance of mitochondria found in any organ tissue, making up 30% of cardiomyocyte cell volume [46, 47].

**Fig. 2:**
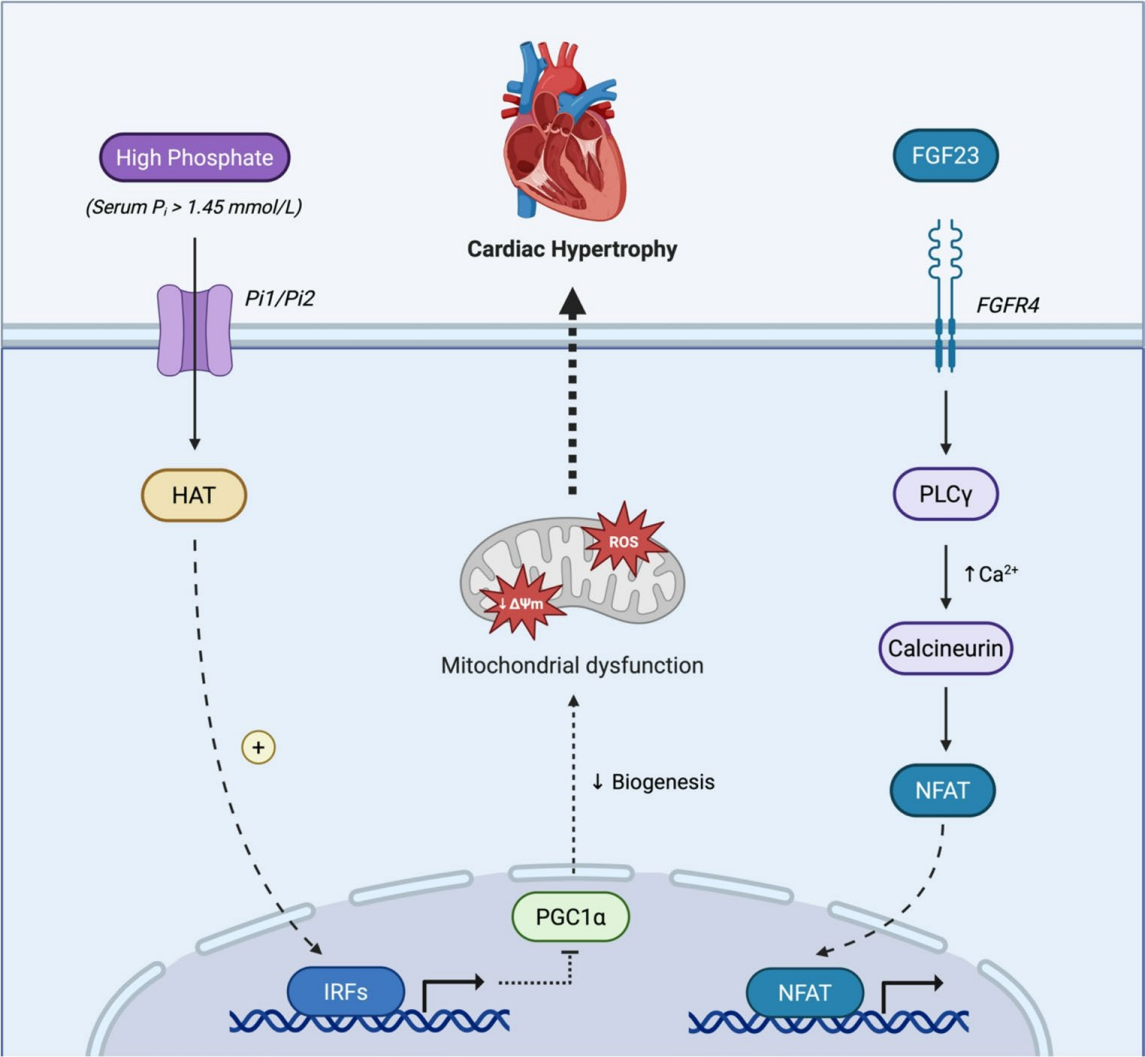
Contribution of high phosphate to CRS 4 associated cardiac hypertrophy. Serum inorganic phosphate (P_i_) enters cardiomyocytes through Pit1 and Pit2. High P_i_ levels cause H3K9 acetylation via HATs, opening the IRF1 promoter and triggering transcription. In turn, IRF1 binds to the promoter region of PGC1α and represses its expression, resulting in mitochondrial dysfunction. Concurrently, high phosphate stimulates the release of FGF23 from osteocytes, which circulates to the heart, binding FGFR4. Recruitment of PLCγ and subsequent Ca²⁺ release activates calcineurin, allowing for rapid nuclear import of NFAT, upregulating hypertrophic genes such as ANP, BNP,β MHC, and RCAN1. These pathways synergistically promote cardiac fibrosis and hypertrophy. Abbreviations: CRS-4 – cardiorenal syndrome type 4, P_i_ – inorganic phosphate, HAT – histone acetyltransferase, IRF – interferon regulatory factor, PCG1a – proliferator-activated receptor gamma co-activator-1-alpha, ROS – reactive oxygen species, FGF23 – fibroblast growth factor 23, NFAT – nuclear factor of activated T-cells, PLCg – Phospholipase C gamma, FGFR4 – fibroblast Growth Factor Receptor 4, ANP – atrial natriuretic peptide, BNP – brain natriuretic peptide, b-MHC – beta-myosin heavy chain, RCAN1 – regulator of calcineurin 1

Mitochondrial biogenesis describes the process by which new mitochondria are produced and is a complex mechanism that requires coordinated regulation of the mitochondrial and nuclear genomes by the transcriptional co-activator peroxisome proliferator-activated receptor gamma co-activator-1-alpha (PGC1α) [10]. Huang et al. demonstrated in a CKD mouse model that high phosphate (HP), a hallmark of CKD, inhibited the expression of PGC1α in cardiomyocytes and subsequently caused LVH [22]. The proposed mechanism (Fig. 2) involves HP-induced interferon regulator factor 1 (IRF1) binding to the promoter region of PGC1α and subsequently inhibiting its expression, leading to dysfunctional mitochondrial energy metabolism and LVH [22]. In addition to this, HP can stimulate the release of FGF23 from osteocytes which then binds to fibroblast growth factor receptor 4 (FGFR4), causing recruitment of phospholipase C gamma (PLCγ), Ca^2+^ release and subsequent calcineurin activation, rapid nuclear import of NFAT, and upregulation of hypertrophic genes [48, 49].

**Fig. 3:**
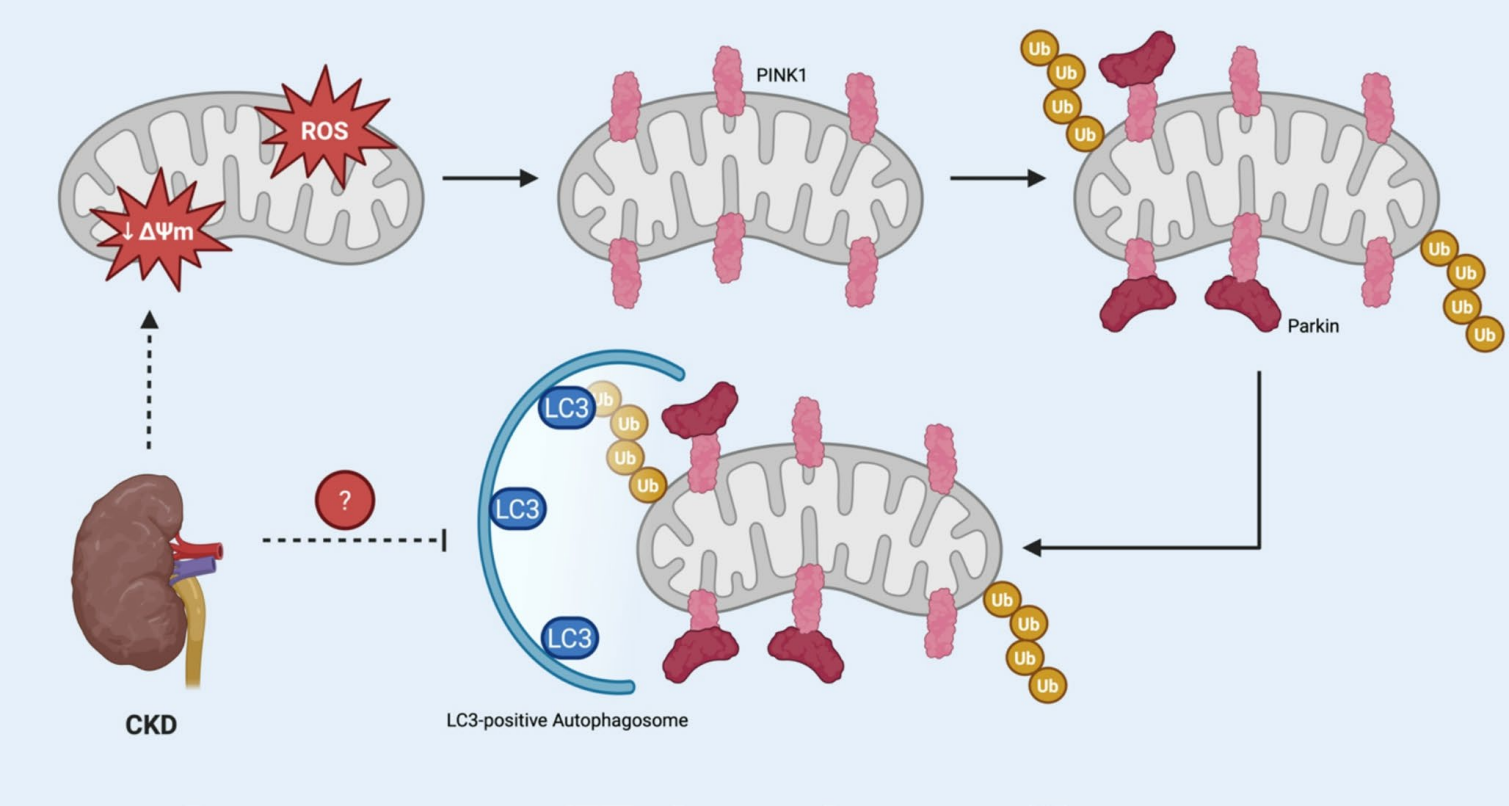
Dysregulation of PINK1/Parkin mediated mitophagy in CRS-4. Under physiological conditions, damaged mitochondria are tagged for removal by mitophagy. This quality control system relies on PINK1 accumulation on depolarised mitochondria and subsequent recruitment of the E3-ligase Parkin. The resulting ubiquitination of OMM proteins causes engulfment by LC3-positive autophagosomes. In CRS-4, despite high levels of mitochondrial dysfunction in cardiomyocytes, dysregulation of the PINK1/Parkin system, for reasons yet to be fully elucidated, prevents mitophagy and perpetuates pro-hypertrophic mitochondrial cGAS-STING and NF-κB signalling. Abbreviations: ROS – reactive oxygen species, PINK1 – PTEN-induced kinase 1, Parkin – E3 ubiquitin ligase, Ub – ubiquitin, LC3 – Microtubule-associated Protein 1 Light Chain 3, CKD – chronic kidney disease, cGAS – cyclic GMP-AMP synthase, STING – stimulator of interferon genes, NF – nuclear factor, OMM – outer mitochondrial membrane

Mitophagy is an autophagic process in which damaged, and thus potentially cytotoxic, mitochondria are recycled. Mitophagy is induced when the mitochondrial membrane potential becomes disrupted and is modulated by E3 ubiquitin ligase (Parkin) And PTEN-induced kinase 1 (PINK1) proteins (Fig. 3) [50]. Ubiquitination of outer mitochondrial membrane proteins causes engulfment of mitochondria by microtubule-associated Protein 1 Light Chain 3 (LC3)-positive autophagosomes. Loss of the mitochondrial membrane potential also triggers the recruitment of PINK1 by Parkin which promotes the clearance of mitochondria via autophagosome degradation [50]. PINK1 and Parkin have also been shown to modulate the cGAS-STING pathway and so disruption of this process can lead to LVH as described above [51]. There are also PINK-independent pathways by which mitophagy can be stimulated, involving activation of mitophagy receptors located on the outer mitochondrial membrane, such as FUN14 domain-containing 1 (FUNDC1), that subsequently promote autophagosome engulfment [52]. Disruption of FUNDC1 signalling has been shown to promote cardiac dysfunction and heart failure [53]. Impairment of cardiac mitophagy has been linked to the development of heart failure in high-fat diet mice [54, 55] and upregulation of mitophagy has been shown to be protective against the progression of heart failure after myocardial infarction [56] as well as the development of atherosclerosis [57, 58]. In CRS-4, despite high levels of mitochondrial dysfunction in cardiomyocytes, dysregulation of the PINK1/Parkin system, for reasons yet to be fully elucidated, prevents mitophagy and so promotes activation of mitochondrial cGAS-STING, NF-κB signalling, and subsequent LVH. Shen et al. demonstrated in a mouse model of CRS-4 that dapagliflozin, a sodium-glucose co-transporter 2 inhibitor (SGLT2i), reduced myocardial injury through inhibition of FUNDC1-dependent mitophagy which suggests that CRS-4 may render FUNDC1-dependent mitophagy defective via the pyruvate kinase isozyme (PKM2)/protein phosphatase (PP1)/FUNDC-1 mitophagy pathway [11]. At which CKD stage defective cardiomyocyte mitophagy occurs is unclear and remains to be fully elucidated.

Mitochondrial dynamics describes the balance between the opposing processes of fission and fusion which allows mitochondria to respond to cellular stress [12]. Fission allows the segregation of dysfunctional mitochondria and subsequent selective elimination of injured mitochondria by mitophagy. Fusion allows mitochondria to maintain integrity in times of stress by combining partially impaired mitochondria and subsequently preventing their excessive clearance. If disequilibrium in mitochondrial dynamics occurs and fission becomes predominant, excess mtROS production and fragmentation can occur [59]. Dynamin-related protein 1 (Drp1) is a cytosolic protein that can be recruited to initiate fission, and excess stimulation can induce increased mtROS production alongside cardiomyocyte injury [12, 59]. The plausibility of a causal link between CKD and perturbed mitochondrial dynamics in cardiomyocytes was demonstrated in a study by Huang et al., which revealed that the uraemic toxin hippurate induces Drp1 mitochondrial fission alongside mtROS production and endothelial dysfunction [12].

**Fig. 4:**
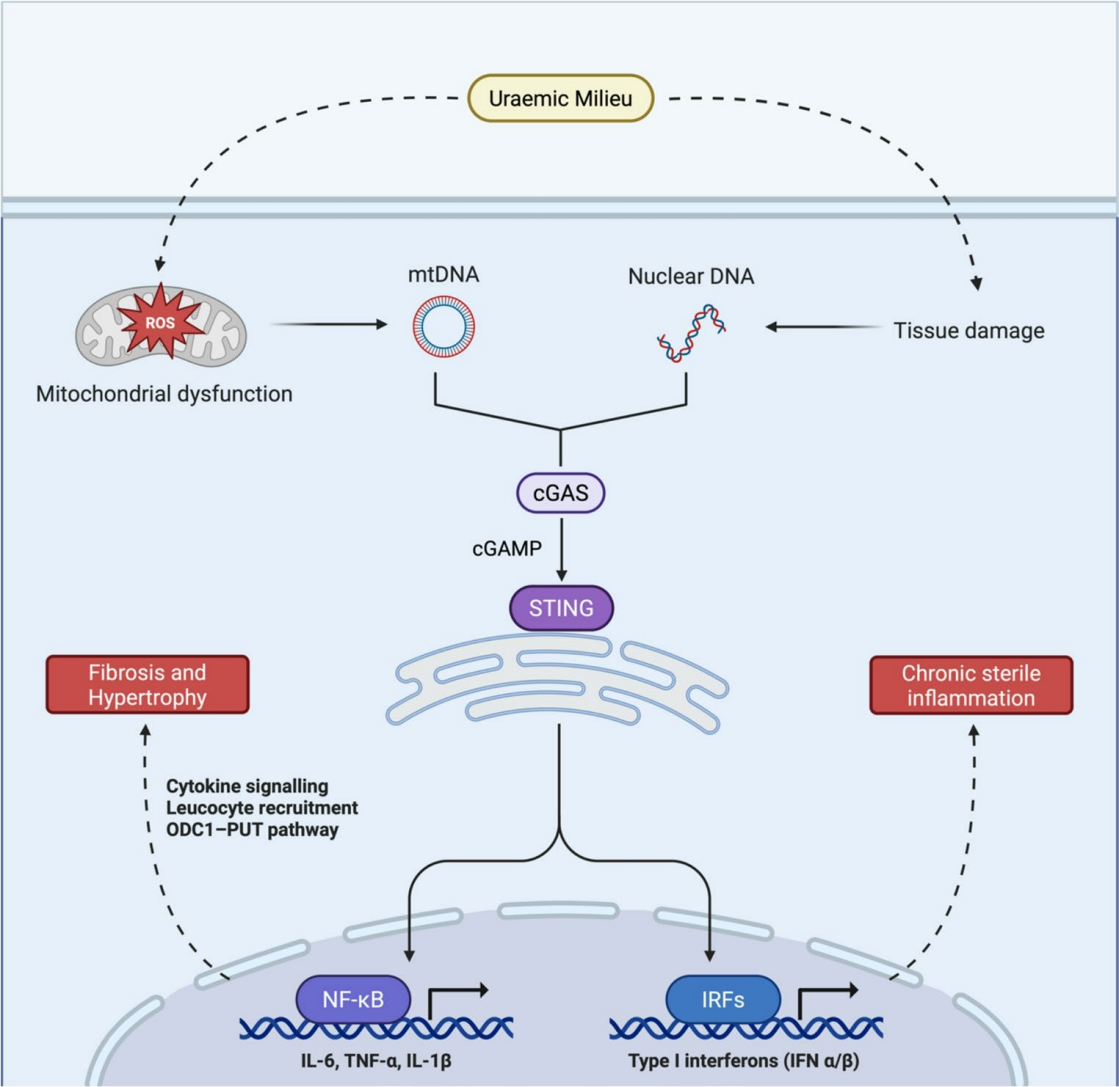
Contribution of the cGAS STING pathway to CRS-4-associated cardiac hypertrophy. The uraemic milieu in CRS 4 induces oxidative stress, causing mitochondrial dysfunction and the release of mtDNA. Binding of mtDNA, alongside dsDNA from tissue damage, by cGAS and subsequent cGAMP synthesis causes activation of STING, which in turn causes nuclear translocation of the transcription factors IRF3 and NFκB, producing type I IFNs (IFN-α/β), and pro-inflammatory cytokines (TNF-α, IL-6, IL-1β) respectively. NF-κB also upregulates ODC1 and the ODC1-PUT pathway. The summative effect of this signalling is cardiac fibrosis and hypertrophy, resulting in progressive heart failure. Abbreviations: mtDNA – mitochondrial DNA, DNA – deoxyribonucleic acid, ROS – reactive oxygen species, cGAS – cyclic GMP-AMP synthase, STING – stimulator of interferon genes, cGAMP – cyclic GMP-AMP, NF- nuclear factor, IFN – interferon, ODC1 – ornithine decarboxylase, PUT – putrescine, IL – interleukin, TNF – tumour necrosis factor, IRF – interferon regulatory factor

The uraemic milieu present in CKD is associated with increases in mtROS production, which can have deleterious effects on cardiomyocytes through the release of mtDNA and subsequent activation of the cGAS-STING-NF*κ*B pathway (Fig. 4) [42, 60]. The release of mtDNA can be sensed by cGAS, causing cyclic GMP-AMP (cGAMP) synthesis, which then activates STING [42, 61]. STING can then cause nuclear translocation of transcription factors IRF3 and NF-*κ*B, causing production of type I IFNs and pro-inflammatory cytokines, respectively [61]. NF-*κ*B can also upregulate the ornithine decarboxylase (ODC1) and the ODC1-putrescine (PUT) pathways [42]. Ultimately, these cause activation of genes involved in cardiac hypertrophy and fibrosis [13, 42]. Moreover, mtDNA is susceptible to oxidative damage and so can acquire mutations which lead to defective biogenesis [62]. This abnormal biogenesis has been linked to increased deletions in mtDNA, which may reflect a replicative advantage conferred on deletion-containing mtDNA, as it has been demonstrated that partially deleted mtDNA can repopulate faster compared to unaltered full-length mtDNA [63]. As this effect compounds, mtDNA deletions may reach a certain threshold whereby cardiomyocytes can no longer function physiologically, inducing further respiratory chain abnormalities and ROS production [62]. The direct mechanism by which the uraemic milieu induces mtROS production in cardiomyocytes remains to be fully understood but may include the elevated release of AngII, transforming growth factor-1β, and endothelin-1 [44]. Moreover, the release of these molecules can activate FGF2, a known promoter of cardiac fibrosis and hypertrophy, and so FGF2 may also be a key mediator of oxidative stress-induced cardiomyocyte damage [44].

## Mitochondria as a Therapeutic Target in CRS-4

As an understanding of the mitochondrial pathways implicated in CRS-4 has emerged, novel treatment strategies that target mitochondria in cardiomyocytes are being explored (Table 2).

**Table 2:** Mitochondrial therapies for CRS 4

Name of therapy	Drug class	Proposed mitochondrial mechanism of action(s)	Reference
Dapagliflozin	SGLT2 inhibitor	Activation of the PKM2/PP1/FUNDC1-mitophagy pathway	[11]
Sacubitril/valsartan (Entresto)	Angiotensin II receptor antagonist	Decrease in mitochondrial swelling via reduced Drp1 expression and increased MFN1 expression	[64]
C-176	STING inhibitor	Inhibition of cGAS-STING pathway	[42]
Ornithine decarboxylase (OCD1)	STING inhibitor	Inhibition of cGAS-STING pathway	[42]
Adeno-associated virus 9 (AAV9)	cGAS inhibitor	Inhibition of cGAS-STING pathway	[69]
N-acetylcysteine (NAC)	Mucolytic agent	Increases mitochondrial GSH levels which modulates mitochondrial redox signalling through redox-sensitive cysteine residues Increases SIRT3 levels, reducing mitochondrial protein lysine acetylation via SOD-2, reducing oxidative stress	[14]
Szeto-Schiller-31 (SS-31)	Mitochondria-targeting antioxidant	Preserves mitochondrial integrity by preventing cardiolipin peroxidation by cytochrome *c* peroxidase Upregulation of SIRT1 and SIRT3 leading to regulation of mitochondrial fission and fusion	[84, 85]
Mitochondrial division inhibitor-1 (Mdivi-1)	Small-molecule Drp1 inhibitor	Inhibits Drp1 – a promoter of mitochondrial fission	[88]
Apocynin	Methoxy-substituted catechol	Inhibition of NADPH oxidation subsequently prevents activation of the ERK1/2 pathway and subsequent upregulation of FGF2	[44]
Stem cell therapy	–	MSC administration causes modulation of PINK1/Parkin mediated mitophagy Transplantation of mitochondria derived from MSCs Delivery of mitochondrial rich extracellular vesicles from autologous stem-cell derived cardiomyocytes	[91–94]
α-Ketoglutarate (AKG)	Tricarboxylic acid metabolite	AKG oxidates NADH to NAD^+^ which is transported to the mitochondria and upregulates SIRT1, regulating autophagy via PINK1 and Parkin	[95, 96]
JQ1	BET protein inhibitor	Modulation of PINK1/Parkin mediated mitophagy	[101]

Abbreviations: CRS-4 – type 4 cardiorenal syndrome, SGLT2 – sodium-glucose cotransporter 2, PKM2 – pyruvate kinase isozyme M2, PP1 – protein phosphatase 1, FUNDC1 – FUN14 domain-containing 1, dynamin-related protein 1, MFN1 – mitofusin 1, cGAS – cyclic GMP-AMP synthase, STING – stimulator of interferon genes, GSH – glutathione, SIRT3 – Sirtuin 3, SOD-2 – superoxide dismutase 2, Drp1 – dynamin-related protein 1, ERK – extracellular signal-regulated kinases, FGF2 – fibroblast growth factor‐2, ROS – reactive oxygen species, PINK1 – PTEN-induced kinase 1, Parkin – E3 ubiquitin ligase, MSC – mesenchymal stem cell, mdivi-1 – mitochondrial division inhibitor-1, NAD – Nicotinamide adenine dinucleotide, AKG – α-Ketoglutarate

As discussed above, excessive production of AngII in CKD may trigger mitochondrial dysfunction, and so AngII receptor blockade (ARB), using sacubitril/valsartan (Entresto), has emerged as a potential therapy to inhibit this damage in CRS-4 and thus mitigate the onset of heart failure. Bigelman et al. studied this approach in a group of Lewis rats in whom CKD was induced by 5/6 nephrectomy [64]. In CKD rats, the authors observed damaged, swollen, and highly disorganised cardiac mitochondria, alongside LVH and interstitial fibrosis. Mitochondrial swelling was associated with increased PARP1 cleavage and cytochrome *c* leakage, which are both markers of cellular apoptosis [65]. There was increased expression of the mitochondrial fusion protein MFN1 and decreased expression of the fission protein Drp1, indicative of increased mitochondrial fragmentation, which was partially salvaged by ARB. However, ARB did not increase total mitochondrial content or change PCG1α expression levels, which suggests that it did not have an impact on *de novo* mitochondrial biogenesis. ARB also did not affect PINK1 levels, suggesting it had a limited impact on mitophagy. Thus, ARB may mitigate the development of CRS-4 through a reduction in mitochondrial swelling, which is predominantly driven by CKD-mediated cardiac hypertrophy as opposed to mitochondrial quality control processes or mitochondrial-induced cell death. Clinical evidence for the use of ARB in CRS-4 is perhaps provided by the UK HARP-III trial which demonstrated that ARB administration in patients with CKD reduced troponin and N-terminal prohormone of brain natriuretic peptide (NTproBNP) levels over 12 months [66]. Moreover, a case report recently published by Cheng et al., in which a patient with CRS-4 received sacubitril/valsartan treatment for 3 years, alongside guideline-directed medical therapy, demonstrated significant improvements in New York Heart Association (NYHA) class alongside improvements in LV ejection fraction [67]. However, this is an isolated case report so should be interpreted with caution. Moreover, no attempt was made to link observed clinical improvements to alterations in mitochondrial processes, so it is not possible to ascertain if the mechanism driving the observed benefits was due to improvements in cardiac mitochondrial function.

Another emerging mitochondrial therapeutic target in CRS-4 is the cGAS-STING pathway. The cGAS-STING pathway can be activated by the uraemic milieu and release of mtDNA and has been linked to the activation of genes which promote LVH [42]. Therefore, inhibition of this pathway provides a mechanism by which LVH can be prevented. Han et al. studied this approach in a CKD mouse model and found that administration of the STING inhibitor C-176 prevented CKD-induced LVH [42]. Moreover, inhibition of ornithine decarboxylase (OCD1) – which is transactivated by the cGAS-STING pathway and an activator of ATP-producing metabolic pathways which promote LVH – using difluoromethylornithine (DFMO) also led to inhibition of CKD-induced LVH. In a transverse aortic constriction (TAC) model of cardiac hypertrophy, Zhou et al. demonstrated that PINK1 deficiency promoted overactivity of cGAS-STING and LVH via the release of mtDNA. STING dependence was illustrated using PINK1/STING double knockout mice [68]. Therefore, PINK1-mediated mitophagy may have a protective role mediated by inhibition of the cGAS-STING pathway which could be targeted therapeutically. Finally, Hu et al. inhibited cGAS using adeno-associated virus 9 in a murine model of heart failure and demonstrated that inhibition of cGAS-STING after TAC led to the preservation of LV function, decreased pathological remodelling, and improved early survival rates [69]. These studies demonstrate that inhibition of cGAS-STING constitutes a promising target in the treatment of CRS-4.

As discussed previously, SGLT2i, specifically dapagliflozin, have been demonstrated to confer a mitoprotective effect [70] which may alleviate myocardial injury in CRS-4 [11]. In a subtotal nephrectomy mouse model of CRS-4, dapagliflozin protected cardiac mitochondria, evidenced by decreased mtROS production, increased ATP production, preserved mitochondrial respiratory complex activity, stabilised the mitochondrial membrane potential, and reduced mitochondrial-induced apoptosis [11]. Mechanistically, dapagliflozin promoted PKM2 expression which subsequently directly interacted with PP1 and FUNDC1, ultimately causing PP1-mediated FUNDC1 phosphorylation and restoration of FUNDC1-dependent mitophagy. Preclinical heart failure studies have also demonstrated that SGLT2i confer mitoprotective effects via upregulating mitochondrial biogenesis and mitochondrial autophagy, inhibiting mitochondrial apoptosis, restoring oxidative phosphorylation, and reducing mtROS production [71–73]. It has also been demonstrated that the cardioprotective effect conferred by acute SGLT2i administration in cardiac ischaemia/reperfusion injury (IRI) may in part be due to attenuation of mitochondrial dysfunction, secondary to decreased mtROS production, decreased swelling, and increased fission [74–76]. Clinically, dapagliflozin has already been demonstrated to significantly reduce cardiovascular death and heart failure hospitalisations in patients with CKD in the DAPA-CKD trial [77], although it remains to be seen if this effect can be attributed to cardiomyocyte mitoprotection.

Reversing the detrimental effects of CKD-induced oxidative stress in cardiac mitochondria provides another avenue for treating CRS-4. Amador-Martínez et al. explored this hypothesis using NAC, an antioxidant modulator of oxidative stress and glutathione (GSH) precursor, in nephrectomy rats [14]. The authors demonstrated that NAC reduced cardiac fibrosis and prevented detrimental changes to cardiac mitochondrial bioenergetics, alongside decreasing mitochondrial H_2_O_2_ production. This was thought to be primarily through increased mitochondrial GSH levels which subsequently modulated mitochondrial redox signalling through redox-sensitive cysteine residues. The authors also found that NAC increased sirtuin 3 (SIRT3) levels, which in turn promoted deacetylation of SOD-2. The resulting increase in SOD-2 enzymatic activity suggests that this pathway represents an additional antioxidant effect of NAC mediated by SIRT3. However, the authors caution that they could not separate the antioxidant effects of NAC from the antihypertensive effect it also conferred. Clinically, acetylcysteine has previously been shown to reduce homocysteine levels, a marker associated with increased oxidative stress, alongside pulse pressure and endothelial function in patients with end-stage renal failure, suggesting NAC may provide a demonstrable clinical benefit [78]. A small crossover randomised controlled trial involving 8 patients with CRS-4 demonstrated that NAC was associated with improved forearm blood flow, although there were no significant changes in NTproBNP levels or NYHA class [79]. These authors hypothesised that the improvement in forearm blood flow was due to an improvement in endothelial function secondary to reduced oxidative stress and proposed that the lack of significant improvements in NTproBNP and NYHA class was due to the small sample size. Nevertheless, this small trial demonstrates that NAC may have a protective effect in CRS-4 via antioxidant pathways, but it remains to be seen whether NAC may specifically confer a significant improvement in mitochondrial function in cardiomyocytes.

Apocynin, a compound extracted from *Picrorhiza kurroa*, is an inhibitor of NADPH oxidase. Liu et al. investigated this compound in CRS-4 and found that apocynin reduced cardiac injury in CRS-4 rats by inhibition of the NOX-dependent oxidative stress-activated ERK1/2 pathway and subsequent inhibition of FGF2 upregulation [44]. However, this effect was not directly linked to mitochondria and the authors found no significant effect of apocynin treatment on mitochondrial superoxide anion production. There is experimental evidence that apocynin may preserve mitochondrial function in diabetic rats [80], but further study is required to elucidate its exact mechanism and whether it directly reduces mtROS production. A mitochondrially targeted derivative of apocynin, mitoapocynin, has been shown to be mitoprotective in neurodegenerative diseases [81, 82]. However, Mahmood et al. demonstrated that mitoapocynin can induce mtROS production in cardiac myoblasts, potentially precluding it as a viable treatment strategy for CRS-4 [83].

SS tetrapeptides are part of a family of aromatic cationic proteins that reside in the inner mitochondrial membrane [84]. SS-31 has been shown to confer a number of mitoprotective benefits including scavenging free radicals, reducing mtROS production, and promoting mitochondrial ATP production [84–86]. Although not directly tested in a model of CRS-4, SS-31 has been shown to preserve cardiomyocyte mitochondrial function in pre-clinical models of pressure overload-induced heart failure [84, 85] and IRI [86]. The mitoprotective mechanism may act via the upregulation of SIRT1 and SIRT3, which have direct interactions with key proteins involved in mitochondrial fission And fusion, including optic atrophy-associated protein 1 [85, 86]. Alternatively, SS-31 may also prevent cytochrome *c*-induced cardiolipin peroxidation, which is associated with the development of heart failure [84, 87].

Mitochondrial division inhibitor-1 (mdivi-1) exerts its mitoprotective effect by inhibiting Drp1-mediated fission. It has been demonstrated to confer a protective effect in a mouse model of CRS-3 by Sumida et al. who found that administration of mdivi-1 prior to renal IRI reduced cardiomyocyte mitochondrial fragmentation via inhibition of Drp1 translocation to the mitochondria and suppression of cytochrome *c* release [88]. Numerous groups have shown mdivi-1 administration to be mitoprotective in cardiomyocytes during IRI in rodents and preserve LV function [59, 88, 89], although it should be noted that this effect was not replicated in a larger, more clinically relevant porcine model of myocardial infarction [90].

Stem cell therapy holds promise as a therapeutic strategy in a range of diseases and, in the context of mitochondrial therapies, has been investigated as a source of exogenous mitochondria which may replenish damaged mitochondria in diseased hearts. There are different approaches that can be taken, including direct administration of mesenchymal stem cells (MSCs) [91, 92], extraction and delivery of mitochondria-rich extracellular vesicles from autologous stem cell-derived cardiomyocytes [93], and direct transplantation of exogenous mitochondria derived from MSCs (MSC-mt) [94]. Although none of these therapies have been directly tested in CRS-4, Ikeda et al. demonstrated that intramyocardial injection of human-derived mitochondria-rich extracellular vesicles restored PGC1α-mediated mitochondrial biogenesis and significantly improved post-MI cardiac function in mice [93]. Thus, administration of mitochondria-rich extracellular vesicles may be a viable therapeutic option. Alternatively, direct transplantation of MSC-mt may confer a therapeutic benefit. Liang et al. demonstrated that direct injection of MSC-mt into a mouse model of MI inhibited cardiac remodelling and apoptosis, reducing mtROS production [94]. Others have also shown that direct administration of MSCs may reduce endothelial dysfunction via modulation of PINK1/Parkin-mediated mitophagy in the setting of DM [91, 92].

Α-Ketoglutarate (AKG), an intermediate of the tricarboxylic cycle, has been demonstrated to promote mitophagy and alleviate cardiac damage in TAC mice models of pressure overload-induced heart failure [95, 96]. An et al. found that AKG administration increased the expression of PINK1 and Parkin mRNA, suggesting that AKG increased mitophagy to clear damaged mitochondria and thus reduced myocardial oxidative stress, ultimately causing decreased LVH, fibrosis, and systolic dysfunction [95]. Yu et al. demonstrated AKG to have a similar effect, which was also attributed to improved mitophagy, alongside inhibition of ferroptosis [96]. Mechanistically, AKG oxidises NADH to NAD^+^ which is subsequently transported to the mitochondria. SIRT1 activity is dependent on NAD^+^ [97] and can regulate autophagy via PINK1 and Parkin.

JQ1 is a selective bromodomain inhibitor that targets BET proteins, notably Bromodomain-containing protein 4 (BRD4) [98]. BET inhibition has been linked to the reversal of pathogenic cardiomyocyte hypertrophy in both TAC-induced models of heart failure [99, 100] as well as diabetic cardiomyopathy [101]. Mu et al. investigated the link between BRD4 and diabetic cardiomyopathy in a diabetic mouse model and demonstrated that overexpression of BRD4 suppresses PINK1/Parkin-mediated mitophagy, causing mitochondrial dysfunction and impairments in cardiac structure and function [101]. JQ1 administration restored PINK1/Parkin-mediated mitophagy and subsequently prevented impairments in cardiac structure and function, which was not seen in mice with *Pink1* deletion, demonstrating a causal pathway linking JQ1 to the restoration of mitophagy. However, a study by Kim et al. which demonstrated BRD4 to have a key role in myocardial contractile function and mitochondrial homeostasis should be noted [102]. The authors engineered a BRD4 knockout mouse and found that loss of BRD4 protein caused downregulation of key mitochondrial genes and genes involved in excitation-contraction coupling. Therefore, any JQ1 therapy should be monitored with caution.

## Conclusion

The pathophysiology of CRS-4 is multifactorial and arises secondary to the uraemic milieu imposed by CKD, alongside homeostatic compensatory mechanisms which cause pressure and volume overload in the heart. Mitochondria have appeared as an emerging link between CKD and subsequent cardiovascular disease, and there is a wealth of pre-clinical evidence that details the mitochondrial processes disrupted in cardiomyocytes during CRS-4. As the understanding of mitochondrial dysfunction in CRS-4 has progressed, new therapeutic avenues have been discovered that may enable the treatment of CRS-4 patients. However, there remains a lack of clinical evidence investigating these new treatments. Real-world clinical trials are required to determine whether mitochondrial targeting offers meaningful clinical benefits to patients with CKD.

## Data Availability

No datasets were generated or analysed during the current study.
